# The potential of soluble programmed death-ligand 1 (sPD-L1) as a diagnosis marker for colorectal cancer

**DOI:** 10.3389/fonc.2022.988567

**Published:** 2022-08-16

**Authors:** Weifang Shao, Yanhua Xu, Suzhen Lin, Junli Gao, Junshun Gao, Hong Wang

**Affiliations:** ^1^ Medical Laboratory Department, ChangXing People Hospital, Hangzhou City, China; ^2^ Hangzhou Cosmos Wisdom Mass Spectrometry Center of Zhejiang University Medical School, Hangzhou City, China

**Keywords:** colorectal cancer, soluble PD-L1 (sPD-L1), lymph node metastasis, sPD-L1, diagnosis marker

## Abstract

Colorectal cancer (CRC) is one of the most significant neoplasms with high morbidity and mortality. Activation of the programmed death protein 1/programmed death ligand 1 (PD-1/PD-L1) signaling pathway results in tumor immune evasion by suppressing the activity of T cells. The correlation of soluble PD-L1 (sPD-L1) in serum/plasma with clinicopathological features, lymph node metastasis, diagnosis and prognosis is less clear. The aim of this study was to investigate the relationship between sPD-L1 and clinicopathological features, and diagnosis potentialof CRC. Three hundred patients with CRC were included in this study. sPD-L1 was measured by ELISA. Pretreatment levels of sPD-L1 were significantly elevated in CRC patient sera compared to healthy control (HC) (*P*<0.001). The median value of sPD-L1 in HC, CRC with non-lymph node metastasis, and CRC with lymph node metastasis were 246.78±50.2pg/mL, 284.12±52.7pg/mL, and 321.31±55.3pg/mL, respectively. ROC analysis of sPD-L1 allowed significant differentiation between HC group and CRC group (lymph node metastasis and non lymph node metastasis (AUC=0.861, 95% CI 0.830-0.887, p<0.001). sPD-L1 is a potential biomarker for the diagnosis of CRC. Multivariate analysis showed that lymph node metastasis and tumor differentiation were independent prognostic factors (all *P*< 0.01), and sPD-L1 was not correlated with the CRC prognosis (*p*>0.05).

## Introduction

Colorectal cancer (CRC) is a significant cause of cancer‐related deaths worldwide, and its incidence and mortality are increasing each year. It accounts for approximately 10% of all annually diagnosed cancers and cancer-related deaths worldwide (1). Despite the improvement in treatment of CRC, cancer recurrence and metastasis remain major causes of therapy failure. The tumor immune suppression and escape play the pivotal roles in progression, recurrence and metastasis of cancerous cells. Especially, the activation of the programmed death protein 1/programmed death ligand 1 (PD-1/PD-L1) pathway was found as one of the key mechanisms in tumor immune evasion (2). Thus, antibody-based immunotherapies blockading the PD-1/PD-L1 signaling pathways in the tumor microenvironment and stimulating the T-cell anti-tumor activity are a promising approach for developing novel tumor therapeutics in routine clinical practices (2–4).

PD-L1, an immune-regulatory molecule, is highly expressed on tumor cells and can be present on activated T and B cells, dendritic cells, and monocytes. Status of PD-L1 expression on tumor cells is connected to the immunotherapy response in some cancer types, and immunohistochemistry (IHC) assay is used to evaluate patients with high likelihood to respond to this targeted therapy. However, in contrast to other tumor entities, PD-L1 IHC expression is not routinely considered in the decision making process on whether or not an immunotherapy targeting PD-1/PD-L1 should be given to a CRC patient. In a recent study from Sato’s group reported that there was no significant difference in overall survival between patients with PD-L1-positive and PD-L1-negative groups (log-rank test, *P* = 0.8218) (5). The expression level of PD-L1 on circulating tumor cells (CTCs) has been applied to the immunotherapy for patients with non small cell lung cancer (6, 7), however CTC enrichment and the immune detection of PD-L1+ CTC remain challenges in routine clinical practices. The level of soluble PD-L1 (sPD-L1) in serum/plasma has been detected from patients with some malignancies including pancreatic cancer, renal cell carcinoma, esophageal cancer, hepatocellular carcinoma, soft tissue sarcomas, metastatic gastrointestinal stromal tumors, and lung cancer (8–16). The elevated sPD-L1 is usually an indicative of poor prognosis of cancer patient. However, the correlation between the levels of sPD-L1 in serum/plasma and the clinicopathologic characteristics in CRC is less clear.

The aim of the present study was to evaluate the changes of sPD-L1 in three cohorts including the HC, CRT without lymph node metastasis, and CRC with lymph node metastasis. The correlation of sPD-L1 levels with clinicopathologic characteristics in CRC is investigated. sPD-L1 as a potential diagnosis biomarker in CRC is also addressed.

## Materials & methods

### Clinical sample collection

#### Therapeutic regimens for CRC patients

This study was approved by the institutional review board of the ChangXing People’s Hospital, ChangXing, Zhejiang, PR. China. To conduct research, permission of the Committee on Bioethics of 2019ZLK-072 dated July 30, 2016, was obtained. All patients gave informed consent to participate in the study. The inclusion criteria were: (1) pathologically diagnosed CRC; and (2) curative surgical resection as the initial treatment. The exclusion criteria were: (1) previous or concurrent cancer; (2) non primary metastatic CRC; (3) patients with heart, liver and kidney insufficiency and serious complications.

The healthy control (HC) was represented by 300 healthy individuals matched for gender and age with the case groups. All donors of the HC group underwent a test of feces for occult blood and fibrocolonoscopy screening. Therefor, the HC group did not include patients with CRC, polyposis, or other defects of the colon or rectum. The case group diagnosed with CRC who underwent surgical resection of primary or metastatic tumors and clinical molecular testing between the years of 2010 and 2015. All patients underwent a general clinical examination at the outpatient stage. The clinical diagnosis was established according to International Classification of diseases, ICD 10. The TNM classification developed by the International Cancer Union was used to classify the stage of cancer and the degree of differentiation. A total of 300 patients were included in this study including CRC without lymph node metastasis (n=150) and CRC with lymph node metastasis (n=150). Blood sampling was carried out in the morning, on an empty stomach, before any treatment. Clinicopathological characteristics including age, gender, tumor size, grades of differentiation, and stage of cancer were obtained from the electronic medical record (**Table 1**).

**Table 1:** Patient characteristics.

	Control Group (*n*=300)	CRC without lymph node metastasis (*n*=150)	CRC with lymph node metastasis (*n*=150)	*p*-value
*Age*, years				
18~65	166 (43.67)	61 (40.67)	66 (44.00)	0.812
>65	169 (56.33)	90 (59.33)	84 (56.00)	
*Gender*				
male	175 (58.33)	89 (59.33)	109 (56.77)	0.607
female	125 (41.67)	61 (40.67)	83 (43.23)	
*Tumor size*				
<5.0cm	–	98 (65.33)	94 (62.67)	0.632
≥5.0cm	–	52 (34.66)	56 (37.33)	
*Tumor differentiation*				
Moderate	–	112 (74.67)	108 (72.00)	0.695
Poor	–	38 (25.33)	42 (28.00)	
*p Stage*				
pT1, pT2	–	52 (34.66)	48 (32.00)	0.596
pT3, pT4	–	98 (65.33)	102 (68.00)	
pN0	–	71 (47.33)	65 (43.33)	0.651
pN1, pN2	–	79 (52.67)	85 (56.67)	

#### Enzyme-linked immunosorbent assay, ELISA

The levels of sPD-L1 in serum were analyzed using Human B7H1/PD-L1 ELISA Kit (ELH-B7H1, RayBiotech, Norcross, GA, USA). All experiments were conducted according to the manufacturer’s instructions. In brief, add 100 µL of standard or twofold-diluted serum sample to each well, and incubated 2.5 h at RT with gentle shaking. Discard the solution and wash 4 times by filling each well with Wash Buffer (300µL) using a multi-channel Pipette. After the last wash, remove any remaining Wash Buffer. Invert the plate and blot it against clean paper towels. Add 100 µL of prepared biotin antibody to each well and incubated 1 h at RT with gentle shaking. Discard the solution, and repeat the wash step. Then, add 100 µL of prepared Streptavidin solution to each well and incubated 45min at RT with gentle shaking. Discard the solution, and repeat the wash step followed by adding 100 µL of TMB One-Step Substrate Reagent to each well and incubating for 30min in the dark at RT with gentle shaking. Add 50 µL of Stop Solution to each well and read at 450nm immediately. Calculate the mean absorbance for each set of duplicate standards and samples, and subtract the average zero standard optical density. Plot the standard curve using Sigma plot software, with standard concentration on the x-axis and absorbance on the y-axis. Finally, the best-fit straight line was drawn through the standard points. The concentrations of sPD-L1 in in serum from CRC and HC were determined according to the standard curve.

#### Follow−up

All CRC patients underwent regular follow-up at our hospital every 3 months for the first 2 years, every 6 months in years 3-5, and annually after that. The primary endpoints were overall survival (OS) and disease-free survival (DFS). OS was calculated from the date of surgery to either the date of death or the last follow-up. DFS was defined as the time from surgery to the time of recurrence (local or distant) or the date of the last follow-up.

#### Statistical analysis

The 95% confidence interval (CI) of all results was calculated. Continuous variables were presented on Mean and Standard Deviation (SD) while categorical variables as Number (*n*) and Percentage (%). Survival analysis was conducted using the Kaplan–Meier method and Cox proportional hazard regression. The multivariate Cox model included all variables with *P*< 0.10 in the univariate model. Fisher’s exact test was used to assess the association of sPD-L1 expression with clinicopathological features. Mann–Whitney U test was performed by SPSS software (version 25.0, IBM, Armonk, NY, USA). Statistical significance was determined when as *P*< 0.05.

## Results

### Patient characteristics

A total of 300 patients were included in the study. By the end of the follow-up period, 196 (65.33%) patients had relapsed, and 139 (46.33%) patients had died of cancer-related causes. The median DFS and OS times for the whole population were 47.8 months (95% CI 37.9-55.2 months) and 53.2 months (95% CI 38.6-58.1 months), respectively. A summary of patients characteristics is shown in **Table 1**.

### sPD−L1 levels in HC and CRC patients

**Figure 1:**
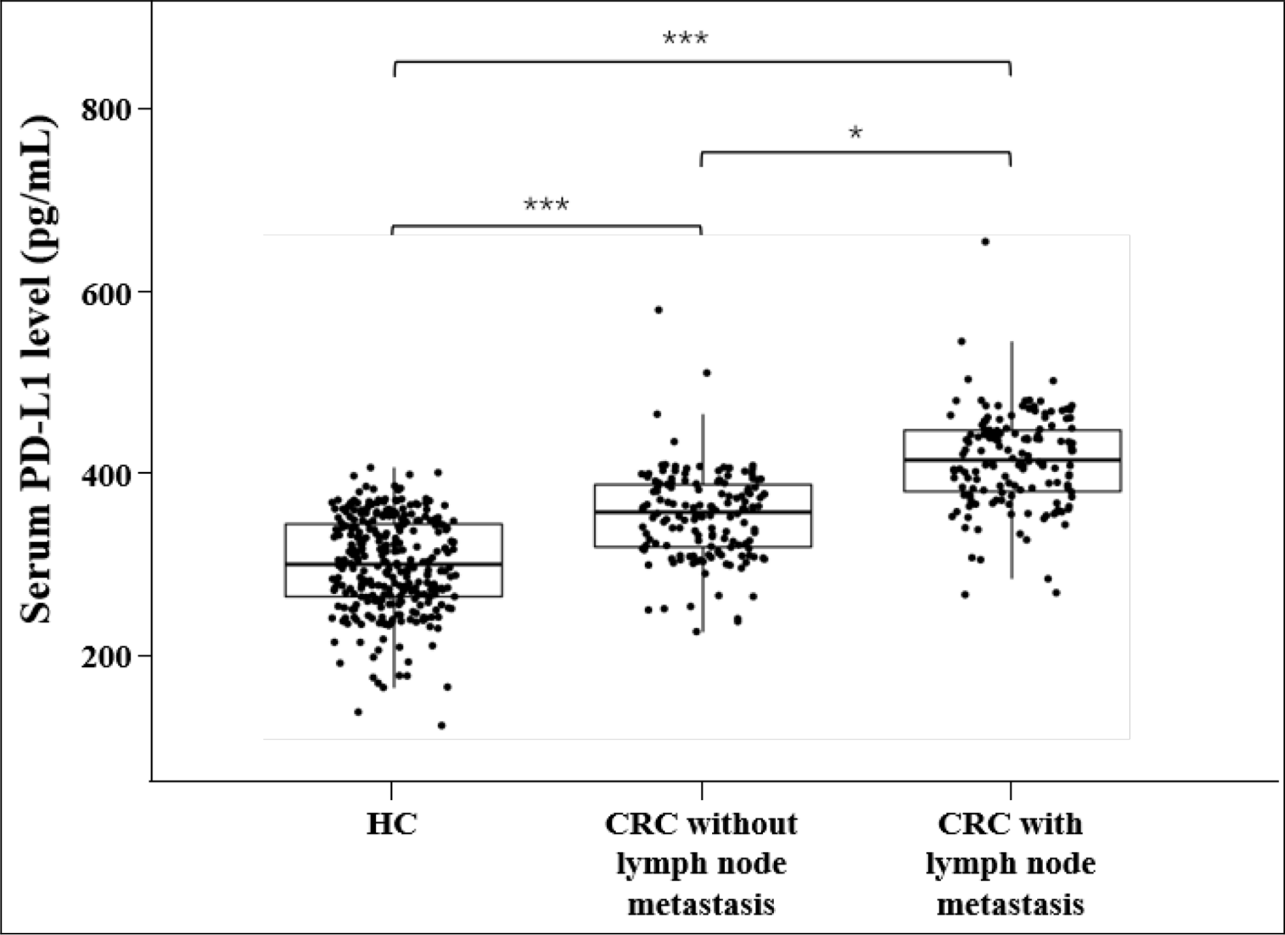
sPD-L1 levels in HC Group and CRC Group *p< 0.05, ***p< 0.001, Mann-Whitney *U* test.

**Figure 2:**
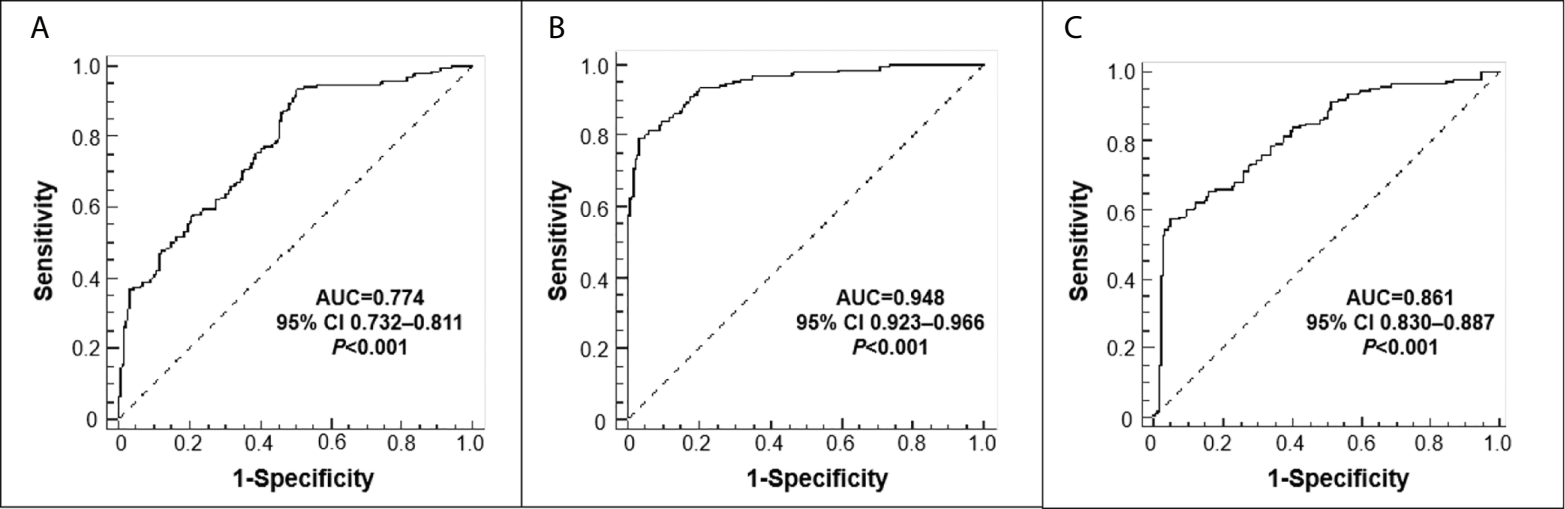
Receiver operating characteristic (ROC) curves. **(A)** HC *versus* CRC without lymph node metastasis; **(B)** HC *versus* CRC with lymph node metastasis; **(C)** HC *versus* CRC (lymph node metastasis+non lymph node metastasis.

The median values of sPD-L1 in HC, CRC without lymph node metastasis and CRC with lymph node metastasis were 246.79 pg/mL, 284.12 pg/mL, and 321.31 pg/mL, respectively. An univariate analysis showed that the level of sPD-L1 was significantly elevated in CRC group compared with the HC group, and sPD-L1 level in CRC group with lymph node metastasis is higher than in the CRC group without lymph node metastasis (all *p*<0.001, **Figure 1**). ROC analysis of sPD-L1 allowed significant differentiation between HC group and CRC group without lymph node metastasis (AUC=0.774, 95% CI 0.732-0.811, *p*<0.001), HC group and CRC group with lymph node metastasis (AUC=0.948, 95% CI 0.923-0.966, *p*<0.001), HC group and CRC group (lymph node metastasis and non lymph node metastasis, AUC=0.861, 95% CI 0.830-0.887, *p*<0.001) as showed in **Figure 2**.

### Correlation between the sPD-L1 levels and clinicopathological features

There was no correlation between the sPD-L1 level and the size of tumor and degree of tumor differentiation (all *p*>0.05, **Table 2**). However, the levels of sPD-L1 was significantly increased with the clinicopathologic staging (all p<0.001, **Table 2**).

**Table 2:** Serum PD-L1 levels and clinicopathological features.

	*n*	PD-L1 [ $\bar{\text{x}}+\text{s}$ ], pg/mL	*p*-value
*Tumor size*			
<5.0cm	192	295.58±52.6	0.087
≥5.0cm	108	301.63±57.2	
*Tumor differentiation*			
Moderate	220	301.37±51.1	0.356
Poor	80	319.53±53.2	
*p Stage*			
pT1, pT2	100	301.02±53.7	0.001
pT3, pT4	200	332.13±55.3	
pN0	136	289.56±51.1	0.001
pN1, pN2	164	331.28±58.1	

### Association of sPD−L1 expression and prognosis of CRC patients

The five-year survival rate of CRC patients enrolled in this study (*n*=300) was 57.67% (173/300), with a median survival time of 51.3 months. A longer median survival time in CRC patients without lymph node metastasis (57.32 months) than in CRC patients with lymph node metastasis (46.12 months) was observed.

The Cox proportional hazards regression analysis showed that the tumor sizes, sPD-L1 levels and clinicopathologic stages were not correlated with the prognosis of CRC (all *p*>0.05, **Table 3**). However, the univariate analysis indicated that the degree of tumor differentiation and lymph node metastasis were statistically associated with the prognosis of CRC (all *p*<0.05, **Table 3**). The multivariate analysis further demonstrated that the tumor differentiation and lymph node metastasis were independent prognostic factors of CRC (all *p*<0.05, **Table 3**).

**Table 3:** Prognosis analysis (Cox proportional hazards model).

	Oddsratio	95% Confidence interval	*p-value*
*Univariate analysis*			
Gender (male/female)	1.652	(0.671~3.576)	0.268
Age, years (<65/≥65)	2.175	(0.903~4.715)	0.125
Tumor size, cm (<5.0/≥5.0)	3.012	(0.867~2.936)	0.316
Tumor differentiation (Moderate/poor)	3.517	(1.035~8.908)	0.018
P-stage (PT1,2/PT3,4)	0.625	(0.095~3.567)	0.517
sPD-L1 levels	1.321	(1.075~2.319)	0.253
Lymph node status (N0/N1,2)	8.762	(2.538~33.672)	0.006
*Multivariate analysis*			
Tumor differentiation (Moderate/poor)	3.603	(0.962~9.106)	0.008
Lymph node status (N0/N1,2)	7.935	(2.182~31.913)	0.005

## Discussion

In present study, we performed a comprehensive analysis of sPD-L1 in a cohort of CRC including the patients with lymph node metastasis (n=150) and the patients without lymph node metastasis (n=150), together with a HC control (n=300). Notably, the examination of sPD-L1 levels in serum revealed that sPD-L1 was significantly elevated in CRC patient compared with the HC control. ROC analysis of sPD-L1 allowed significant differentiation between HC group and CRC group. Thus, our observations have the implication that sPD-L1 may be served as a potential biomarker for the diagnosis of CRC, together with other markers tested in currently clinical practices including CEA, CA19-9 and CA24-2 (14). We didn’t identify the prognosis value of sPD-L1 in the present study although a few reports had addressed the association of sPD-L1 with the prognosis of patients, including the studies with contrasting result in lung cancer (6, 7) and no correlation in gastric cancer (17), worse prognosis in aggressive renal cell carcinoma and shorter survival in multiple myeloma and diffuse large B-cell lymphoma (9, 18). The association between serum sPD-L1 and prognosis of patient with CRC needs to be further investigated by integrating sPD-L1 levels with key clinicopathological features including the status of lymph node metastasis, clinicopathologic stage, and tumor differentiation. Our results also demonstrated that the level of sPD-L1 is higher in CRC patients with lymph node metastasis compared to CRC patients without lymph node metastasis. We found that sPD-L1 isn’t associated with clinical characteristics such as age, gender, tumor size and tumor differentiation. sPD-L1 is more a general marker of an inflammatory status than just a marker of active, immuno-suppressive cancer cells. It reflects the function of PD-L1 expressed in inflamed tissues. However, sPD-L1 contributes to immune regulation together with PD-L1 on tumor cells.

Further, we identified that CRC patients without lymph node metastasis had better five-year survival rate and median survival time in comparison with the lymph node metastasis regardless the tumor size, clinicopathologic stage and sPD-L1 level. To our best knowledge, most of the published works primarily addressed the prognostic relevance of PD-L1, whereas little is known about their predictive value as well as their relationship with molecular genetic alterations in CRC. However, the genetic alterations of PD-L1 have been observed in diffuse large B-cell lymphoma (DLBCL). Dr. Shipp found that chromosome 9p24.1/PD-L1/PD-L2 alterations increase the abundance of the PD-L1 and PD-L2, and their further induction through Janus kinase 2 signal transducers and activators of transcription signaling in classical Hodgkin lymphomas (19). Dr. Pan-Hammarström reported that genetic alterations in the PD-L1/PDL-2 locus are mainly associated with the non-GCB subtype of DLBCL, and translocations between PD-L1 and the IGH locus represent a genetic mechanism of PD-L1 overexpression in DLBCL (20). In a recent study, SP140 was identified as a novel translocation partner for PD-L1, and a new inversion was detected between PD-L1 and PD-L2, both leading to the upregulation of PD-L1 expression in DLBCL (21). Dr. Ogawa and co-workers revealed that a high frequency of PD-L1/PD-L2-involving genetic aberrations was observed in Epstein-Barr virus (EBV)-positive lymphomas (22%), however the frequency was much lower in EBV-negative lymphomas regardless of histology type (5%), suggesting a potential role of detecting PD-L1/PD-L2-involving lesions for these lymphomas to be effectively targeted by immune checkpoint blockade (22). We didn’t examine the genetic alterations of PD-L1 in this study, and plan to identify the genetic alterations of PD-L1/PD-L2 in CRC in our next project.

The identification of patients that are likely to receive a benefit from PD-1/PD-L1 inhibition therapy remains a challenge. The lack of detection of common CRC related genes including EGFR, KRAS, BRAF and p53 in tumor together with CEA, CA19-9 and CA24-2 in serum is the limitation of present study.Our future work will focus on the integration of sPD-L1 level in serum and PD-L1 expression on tumor cells in the separate cohorts to future investigate the roles of sPD-L1 on diagnosis for early detection of CRC, prediction for response to the therapy, risk of lymph node metastasis, and prognosis of patients. The examination of CRC related genes and common serum biomarkers as mentioned above will also be taken to support new findings. The genetic characteristics of PD-1/PD-L1/L2 in CRC also need to be further investigated.

## Data availability statement

The original contributions presented in the study are included in the article/supplementary material. Further inquiries can be directed to the corresponding authors.

## Ethics statement

This study was approved by the institutional review board of the ChangXing People Hospital, ChangXing, Zhejiang, PR. China. To conduct research, permission of the Committee on Bioethics of 2019ZLK-072 dated July 30, 2016, was obtained. All patients gave informed consent to participate in the study.

## Author contributions

WS, YX, SL, HW: protocol/project development. JunliG, YX: data collection and management. HW, WS: data analysis. HW: manuscript writing/editing. All authors contributed to the article and approved the submitted version.

## Acknowledgments

The authors are grateful to Linzhen Song for excellent assistance in data analysis.

## Conflict of interest

The authors declare that the research was conducted in the absence of any commercial or financial relationships that could be construed as a potential conflict of interest.

## Publisher’s note

All claims expressed in this article are solely those of the authors and do not necessarily represent those of their affiliated organizations, or those of the publisher, the editors and the reviewers. Any product that may be evaluated in this article, or claim that may be made by its manufacturer, is not guaranteed or endorsed by the publisher.
